# Factorial Optimization of CoCuFe-LDH/Graphene Ternary Composites as Electrocatalysts for Water Splitting

**DOI:** 10.1021/acsami.4c10870

**Published:** 2024-09-12

**Authors:** Daniele Alves, Rafael A. Moral, Darshana Jayakumari, Eithne Dempsey, Carmel B. Breslin

**Affiliations:** † Department of Chemistry, Maynooth University, Maynooth, Co. Kildare Ireland, W23 F2H6; ‡ Department of Mathematics and Statistics, Maynooth University, Maynooth, Co. Kildare Ireland, W23 F2H6; § Hamilton Institute, Maynooth University, Maynooth, Co. Kildare Ireland, W23 AH3Y; ∥ Kathleen Lonsdale Institute, Maynooth University, Maynooth, Co, Kildare Ireland, W23 F2H6

**Keywords:** Layered double hydroxides, electrocatalysts, graphene, factorial design, oxygen evolution reaction, hydrogen evolution reaction

## Abstract

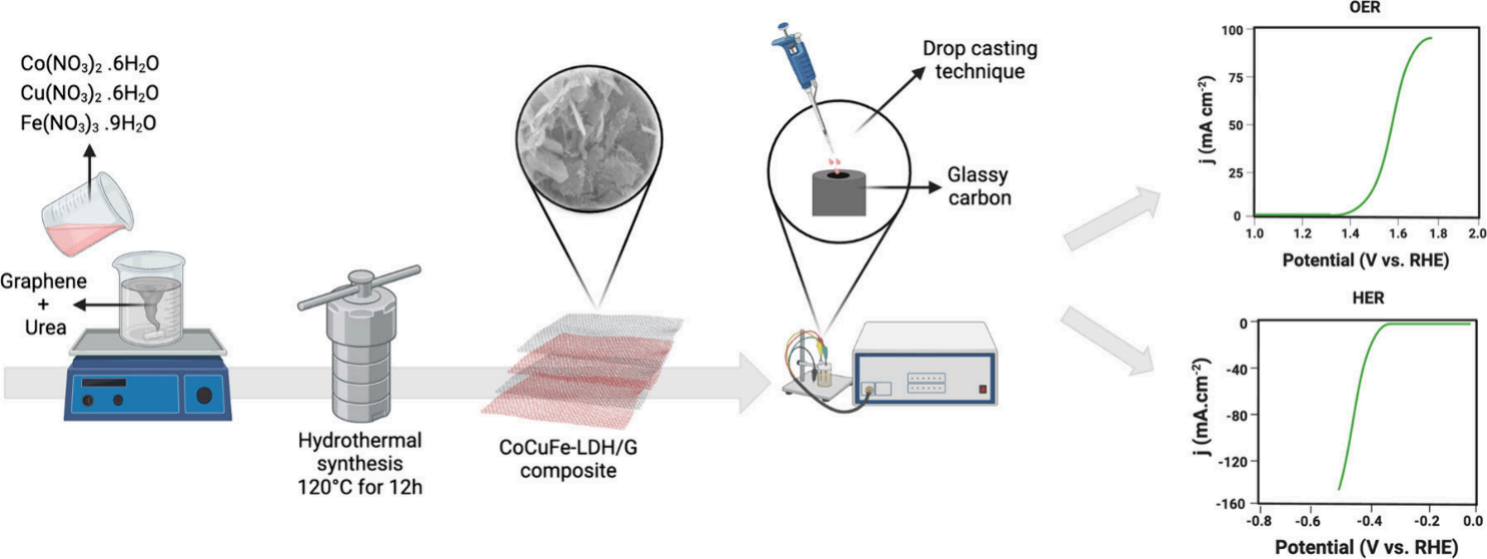

The layered double hydroxides (LDHs) have demonstrated significant potential as non-noble-metal electrocatalysts for the hydrogen evolution reaction (HER) and oxygen evolution reaction (OER). Their unique compositional and structural properties contribute to their efficiency and stability as catalysts. In this study, CoCuFe-LDH composites were grown on graphene (G) via a cost-effective and straightforward one-step hydrothermal process. A 2-level full-factorial model was employed to determine the impact of Co (1.5, 3, and 4.5 mmol) and graphene (10, 30, and 50 mg) concentrations on the onset potential of OER and HER, which were the chosen response variables. OER and HER activity variabilities were assessed in triplicate using Co_[3]_Cu_[3]_Fe_[3]_-LDH/G_[30]_ (central point), which were determined at 0.01% and 0.02%, respectively. Statistical analyses demonstrated that Co_[4.5]_Cu_[3]_Fe_[3]_-LDH/G_[10]_ and Co_[1.5]_Cu_[3]_Fe_[3]_-LDH/G_[10]_ showed the lowest onset potential at 1.52 V and −0.32 V (V vs RHE) for the OER and HER, respectively, suggesting that a high cobalt concentration enhances OER performance, while optimal HER catalysis was achieved with lower cobalt concentrations. Moreover, the trimetallic composites exhibited good stability with negligible loss of catalytic activity over 24 h.

## 1 Introduction

The increasing energy demand, driven by industrialization and global population growth, has increased the focus on developing clean and sustainable energy sources.^1^ Hydrogen (H_2_) has emerged as a promising alternative to carbon-based fuels due to its low cost, higher calorific value, and absence of pollution emissions.^2^ Electrochemical water splitting has recently emerged as a highly promising method for efficient hydrogen production. In this process, the hydrogen evolution reaction (HER) occurs at the cathode, while the oxygen evolution reaction (OER) takes place at the anode during water electrolysis.^3,4^ Addressing the urgent need for large-scale hydrogen fuel production through water electrolysis requires the exploration of new catalysts capable of efficiently catalyzing both the HER and the OER.^5^ Water splitting is a crucial process in the generation of hydrogen, a clean and sustainable fuel. Water electrolysis entails the decomposition of water into hydrogen and oxygen via an applied external electric field.^6^ The advancement of efficient catalysts is crucial for improving the kinetics of water splitting, thereby making the process economically feasible for large-scale energy storage and conversion.^7^

Currently, noble metal oxides, such as iridium and ruthenium oxides, have proven to be effective electrocatalysts for OER, while platinum serves as an efficient electrocatalyst for HER.^8,9^ Despite their notable performance, the elevated costs and restricted availability of these precious metals pose significant barriers to widespread industrial adoption. Consequently, there is an urgent need to develop nonprecious and abundantly available high-performance electrocatalysts for both HER and OER to facilitate sustained overall water splitting.^10,11^ 2D layered double hydroxide (LDH)-based materials are recognized as promising catalyst candidates for energy storage and conversion devices due to their advantageous features, including a lamellar structure, facile synthesis, abundance of earth elements, tunable chemical composition, increased surface area, and exchangeable interlayer anions.^12,13^ Among LDH-based nanostructures, cobalt (Co)-based catalysts emerge as potential alternatives to noble-metal catalysts in water-splitting applications, attributed to the high redox potential of Co species, and stability in both acidic and basic environments.^14,15^

Despite the capability of Co-based LDH to catalyze the HER and OER, it encounters challenges in sluggish water dissociation kinetics during overall water splitting, resulting in unsatisfactory electrocatalytic stability.^14,16^ The synthesis of Co-based LDH nanostructures presents a viable approach to enhance catalytic performance through synergistic effects among different components.^17^ Additionally, the incorporation of earth-abundant copper in water splitting, in combination with various catalyst compositions, holds promise for the replacement of noble metals in commercial hydrogen generation. Copper (Cu)-based materials exhibit high electrical conductivity, the CuFe-LDH has been reported as an interesting electrocatalyst since iron (Fe) can enhance the reactivity in OER and HER processes.^18,19^ Moreover, in binary LDHs, introducing a third metal ion has been shown to change electronic structure and improve conductivity; hence, providing more active sites and facilitating a fast electron transfer process.^20,21^ Accordingly, doping a new active atom into CoFe-LDHs is expected to accelerate the intrinsic OER and HER activities.^22^ Cu can be easily doped into the CoFe-LDH host since the atomic radius of Cu^2+^ is close to that of Co^2+^. Additionally, recent studies have shown that Cu can effectively optimize the distortion degree and electron configuration of bimetallic LDHs, reducing the adsorption energy barrier and thereby enhancing OER and HER activities.^23,24^ Therefore, the combination of Co, Cu and Fe forming CoCuFe-LDH holds great potential for advancing water splitting.

However, LDHs as electrocatalysts are limited by their poor electronic conductivity. A strategy to enhance these limitations is to combine them with other materials that provide better conductivity and stability.^25,26^ Graphene, consisting of a single layer of carbon atoms in a hexagonal lattice, provides an excellent supporting matrix for CoCuFe-LDH due to its exceptional electronic conductivity and high surface area, facilitating efficient electron transfer during the water-splitting reaction.^27,28^ The integration of graphene with CoCuFe-LDH not only stabilizes the LDH structure but also promotes faster charge transport, improving the overall catalytic activity.^29,30^

Therefore, in this work, we aimed to investigate the concentrations of cobalt and graphene using statistical analysis to prepare CoCuFe-LDH/graphene composites with improved OER and HER activities via a cost-effective and facile one-step hydrothermal process. The structural, morphological, and electrochemical properties of the composites were systematically studied and compared with their binary counterparts. This novel approach, employing statistical analysis, facilitates the more efficient preparation of integrated materials, ensuring improved OER and HER performance to address critical energy challenges. In addition, as far as we are aware, CoCuFe-LDH/graphene composites have not been applied as electrocatalysts for water splitting nor have they been previously documented for any other application within the scientific literature.

## 2 Material and Methods

### 2.1 Chemicals and Preparation of Co_[m]_Cu_[3]_Fe_[3]_-LDH/G_[n]_ Composites

Cobalt(II) nitrate hexahydrate (Co(NO_3_)_2_·6H_2_O), copper(II) nitrate hexahydrate (Cu(NO_3_)_2_·6H_2_O), iron(III) nitrate nonahydrate (Fe(NO_3_)_3_·9H_2_O), urea (NH_2_CONH_2_), and graphene nanoplatelets (G) were all purchased from Sigma-Aldrich, UK. A ruthenium oxide rod electrode (99.9%) was purchased from GoodFellow Metals, UK.

The Co_[m]_Cu_[3]_Fe_[3]_-LDH/G_[n]_ composites were synthesized via a hydrothermal process, employing varied molar ratios of Co to Cu and Fe (0.5:1:1, 1:1:1, and 1.5:1:1), with *m* = 1.5, 3, and 4.5 mmol, along with different amounts of graphene nanoplatelets (*n* = 10, 30, and 50 mg). The investigation aimed to understand the impact of these factors on the OER and HER activities. The ratio of Co, Cu, and Fe was selected based on established findings in the literature, where these specific proportions have been demonstrated to optimize the structural and electrochemical performance of the resulting composite. By adhering to these ratios, the aim was to leverage the synergistic effects of these metals to enhance the material’s overall functionality. In the experimental design, a 2-level full factorial design with a central point was employed, as detailed in Table 1, with three replicates. Initially, a specific quantity of graphene nanoplates was dispersed in a 10 mL mixture comprising equal parts of deionized water and ethanol, followed by sonication for 20 min. A second solution was prepared by dissolving a certain amount of Co(NO_3_)_2_, 3 mmol of Cu(NO_3_)_2_, and 3 mmol of Fe(NO_3_)_3_ in 20 mL of deionized water. Simultaneously, 1.5 g of urea was dissolved in 10 mL of deionized water to form a third solution. These three solutions were combined and stirred for 1 h, and the resulting homogeneous solution was then placed in a 100 mL Teflon-lined stainless-steel autoclave and maintained at 120 °C for 12 h. The Co_[m]_Cu_[3]_Fe_[3]_-LDH/G_[n]_ composites were subsequently collected, washed multiple times with ethanol and deionized water, and dried at 60 °C for 18 h. For comparative purposes, a series of control materials with distinct compositions, including CoCuFe-LDH, CuFe-LDH/G, CoCu-LDH/G, and CoFe-LDH/G, were synthesized using the same method.

**Table 1:** Factors and Levels for the Optimization of the Co_[m]_Cu_[3]_Fe_[3]_-LDH/G_[n]_ Composites

**Composites**	**1stfactor [Co]**	**2ndfactor [G]**	**[Co] (mmol)**	**[G] (mg)**
Co_[1.5]_Cu_[3]_Fe_[3]_-LDH/G_[10]_	–1	–1	1.5	10
Co_[4.5]_Cu_[3]_Fe_[3]_-LDH/G_[10]_	+1	–1	4.5	10
Co_[1.5]_Cu_[3]_Fe_[3]_-LDH/G_[50]_	–1	+1	1.5	50
Co_[4.5]_Cu_[3]_Fe_[3]_-LDH/G_[50]_	+1	+1	4.5	50
Co_[3]_Cu_[3]_Fe_[3]_-LDH/G_[30]_	0	0	3	30

### 2.2 Materials Characterization

The optimized Co_[m]_Cu_[3]_Fe_[3]_-LDH/G_[n]_ and the corresponding LDH without graphene, Co_[m]_Cu_[3]_Fe_[3]_-LDH, were selected for further investigations through characterization and electrochemical measurements. The morphologies of the samples were examined using a field emission scanning electron microscope (FE-SEM, Hitachi, S-4800). Elemental information was obtained by energy-dispersive X-ray spectroscopy (EDX, Oxford Instrument INCAz-act ESX system), while X-ray photoelectron spectroscopy (XPS) (Kratos AXIS ULTRA spectrometer) was employed to determine the chemical state of the elements. Crystal structure analysis was conducted using X-ray diffraction (XRD) with a Powder X-ray P-XRD, PANalytical, X’Pert-PRO MPD system. Additionally, Fourier transform infrared spectroscopy (FTIR, Nicolet iS50 FTIR spectrometer) was utilized to analyze the composites.

### 2.3 Electrochemical Characterization

The electrocatalytic activities for the OER and HER of the LDH catalysts were assessed on a glassy carbon electrode (GCE) with a 3 mm diameter. The GCE substrate underwent polishing using Akasol diamond suspensions of 6 and 1 μm sizes on an Aka–Napel microcloth, followed by thorough washing with deionized water, sonication in deionized water, and air drying. The LDH was prepared as an ink by blending 3 mg of LDH with 0.5 mL of deionized water and 0.5 mL of ethanol. After 10 min of sonication, a homogeneous ink was obtained and applied to the GCE through drop casting, maintaining the LDH electrocatalyst loading at approximately 84.9 μL cm^–2^. Electrochemical tests were performed in a three-electrode cell containing 1 M KOH (pH 13.6), utilizing the LDH-modified GCE, a mercury/mercury oxide (Hg/HgO) reference electrode, and a high surface area platinum counter electrode. Linear sweep voltammetry (LSV) was employed as the electrochemical technique at a scan rate of 5 mV s^–1^. All potentials were converted to the relative hydrogen electrode scale (*E*_RHE_ = *E*_Hg/HgO_ + 0.0924 V + 0.059 × pH) and corrected for *iR* drop, while current density was calculated using the geometric surface area of the GCE. Electrochemical impedance spectroscopy (EIS) data were collected across a frequency range from 1 × 10^6^ to 0.007 Hz using fixed potentials at 1.59 V and −0.41 V, which correspond to 10 mA cm^–2^ for OER and HER, respectively. The collected data were fitted to an equivalent circuit, and the charge transfer resistance was determined. Furthermore, the stability of the selected trimetallic LDH, Co_[m]_Cu_[3]_Fe_[3]_-LDH/G_[n]_, was investigated over 24 h at a fixed potential corresponding to a current density of 10 mA cm^–2^.

### 2.4 Statistical Analysis

Linear models were fit to the OER and HER response variables, including replicate (factor with three levels) and a linear response surface over Co and G concentrations in the linear predictor for the mean. The significance of the interaction between Co and G concentrations was assessed via F-tests. Contour plots were produced to display the predicted OER and HER values over a smooth 2D grid for Co and G concentrations. Goodness-of-fit was assessed through half-normal plots with a simulated envelope.^31^ This technique consists of plotting ordered model diagnostics (e.g., residuals) in absolute value versus the expected order statistics of a half-normal distribution. The added simulated envelope is such that if most points fall inside the envelope, then the data is a plausible realization of the fitted model, i.e., the model’s distributional assumptions are met. Contour plots were produced using ggplot2.^32^ All analyses were carried out in R.^33^ All R scripts and data are available at < https://www.github.com/daanielealves/potential_analysis>.

## 3 Results and Discussion

### 3.1 Co_[m]_Cu_[3]_Fe_[3]_-LDH/G_[n]_ Optimization for OER and HER

**Figure 1:**
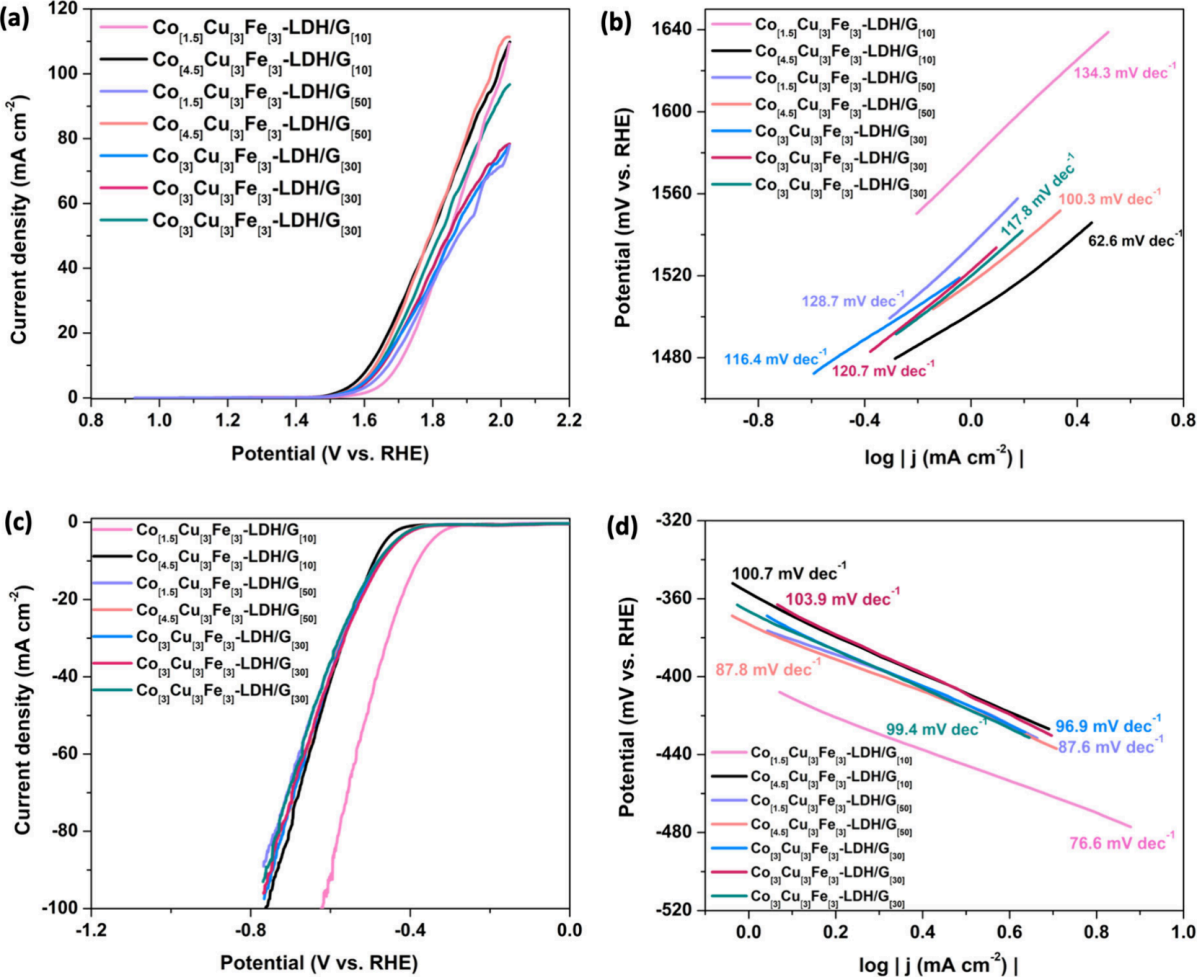
Polarization curves of the samples prepared with different molar ratios of Co(NO_3_)_2_ and amounts of graphene for (a) OER and (b) derived Tafel slopes; (c) HER and (d) derived Tafel slopes.

The Co_[m]_Cu_[3]_Fe_[3]_-LDH/G_[n]_ electrocatalysts synthesized were evaluated for the OER and HER performance through linear sweep voltammetry (LSV) in a conventional three-electrode configuration within a 1.0 M KOH electrolyte at room temperature. The LSV polarization curves and Tafel slope for the OER are illustrated in Figure 1(a) and Figure 1(b), respectively. It can be observed that Co_[4.5]_Cu_[3]_Fe_[3]_-LDH/G_[10]_ exhibited outstanding catalytic activity with the lowest onset potential (1.52 V) and Tafel slope (62.6 mV dec^–1^), demonstrating that an optimal electrocatalyst for the OER is achieved with a high concentration of cobalt (4.5 mmol) and a low concentration of graphene (10 mg). This trend may be due to the good catalytic activity of cobalt, and a higher concentration of this element in the composite provides more active sites for OER, leading to a lower onset potential. Graphene, which is an excellent electrical conductor, ensures efficient electron transfer during the electrocatalytic process. In this context, a lower concentration may be favored to maintain optimal conductivity without compromising the catalytic activity of cobalt. Therefore, the synergy between the catalytic activity of cobalt and the conductive properties of graphene in the specified concentration ratios contributes to the observed superior performance of the Co_[4.5]_Cu_[3]_Fe_[3]_-LDH/G_[10]_ composite as an electrocatalyst for the OER. LSV was conducted again to optimize the composites for HER, the polarization curves and Tafel slope are shown in Figure 1(c) and Figure 1(d), respectively. Co_[1.5]_Cu_[3]_Fe_[3]_-LDH/G_[10]_ composite demonstrates remarkable catalytic efficacy, demonstrating the lowest onset potential (−0.32 V) and Tafel slope (76.6 mV dec^–1^) compared to the other composites. This underscores the attainment of an optimal electrocatalyst for HER characterized by a reduced cobalt concentration and again a lower proportion of graphene. In both the OER and HER, an excessive concentration of graphene could result in the agglomeration of graphene sheets,^34^ leading to diminished dispersion of transition metal centers and a subsequent reduction in active catalytic sites.^35^

Interestingly, higher concentrations of Co are beneficial in the OER, while lower concentrations are more effective in the HER. This may be related to the conversion of Co to the Co-OOH phase (Co^3+^), which is considered the active electrocatalyst in the OER. As there is an equilibrium between the Co^2+^ and this Co^3+^ oxyhydroxide, higher concentrations of Co are beneficial in maintaining a sufficient concentration of the Co^3+^ oxyhydroxide. Moreover, the Co^3+^ plays a critical role in stabilizing intermediate species formed during OER, resulting in enhanced catalytic activity and efficiency.^36^ Conversely, during the HER, the Co remains in the Co^2+^ oxidation state and a relatively lower concentration is beneficial because it helps maintain an optimal balance of active sites, while reducing the likelihood of oversaturation that could reduce catalytic efficiency.^37^

Additionally, to validate the reproducibility of the OER and HER utilizing the synthesized composites, experiments were conducted in triplicate, and the resultant standard deviations are presented in Table 2. The low standard deviations (<0.05) reflect the consistency and reliability of the experimental methodology employed, providing confidence in the reproducibility of the OER and HER employing the synthesized composites.

**Table 2:** Mean Onset Potential (± standard deviation) for the Synthesized Electrocatalysts

**Catalyst**	**Onset potential (V)**
**OER**	
Co_[1.5]_Cu_[3]_Fe_[3]_-LDH/G_[10]_	1.60 ± 0.012
Co_[4.5]_Cu_[3]_Fe_[3]_-LDH/G_[10]_	1.52 ± 0.006
Co_[1.5]_Cu_[3]_Fe_[3]_-LDH/G_[50]_	1.57 ± 0.006
Co_[4.5]_Cu_[3]_Fe_[3]_-LDH/G_[50]_	1.54 ± 0.012
Co_[3]_Cu_[3]_Fe_[3]_-LDH/G_[30]_	1.56 ± 0.006
**HER**	
Co_[1.5]_Cu_[3]_Fe_[3]_-LDH/G_[10]_	–0.32 ± 0.006
Co_[4.5]_Cu_[3]_Fe_[3]_-LDH/G_[10]_	–0.42 ± 0.010
Co_[1.5]_Cu_[3]_Fe_[3]_-LDH/G_[50]_	–0.42 ± 0.012
Co_[4.5]_Cu_[3]_Fe_[3]_-LDH/G_[50]_	–0.40 ± 0.006
Co_[3]_Cu_[3]_Fe_[3]_-LDH/G_[30]_	–0.42 ± 0.012

The results from the regression analyses are presented in Table 3. For both OER and HER, there was a significant interaction between Co and G (*F*_1,9_ = 34.09 and *F*_1,9_ = 34.96, respectively, *p* < 0.001). The estimated linear equations are (averaging across replicates):
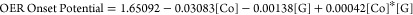
1
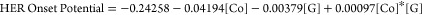
2Here, [Co] is the concentration of cobalt, [G] represents the amount of graphene, and the term [Co]*[G] indicates the interactions between Co and G.

**Table 3:** Summary of the Linear Models Fitted to the OER and HER Data

**Response**	**Effect**	F-test statistic*	***p*-value**	**Adj. *R***^**2**^
OER	Replicate	2.30	0.156	0.93
	Co	165.00	<0.001	
	G	1.36	0.273	
	Co × G	34.09	<0.001	
HER	Replicate	0.09	0.914	0.81
	Co	15.10	0.004	
	G	12.58	0.006	
	Co × G	34.96	<0.001	

* Replicate is associated with 2 and 9 d.f.; all other effects associated with 1 and 9 d.f.

**Figure 2:**
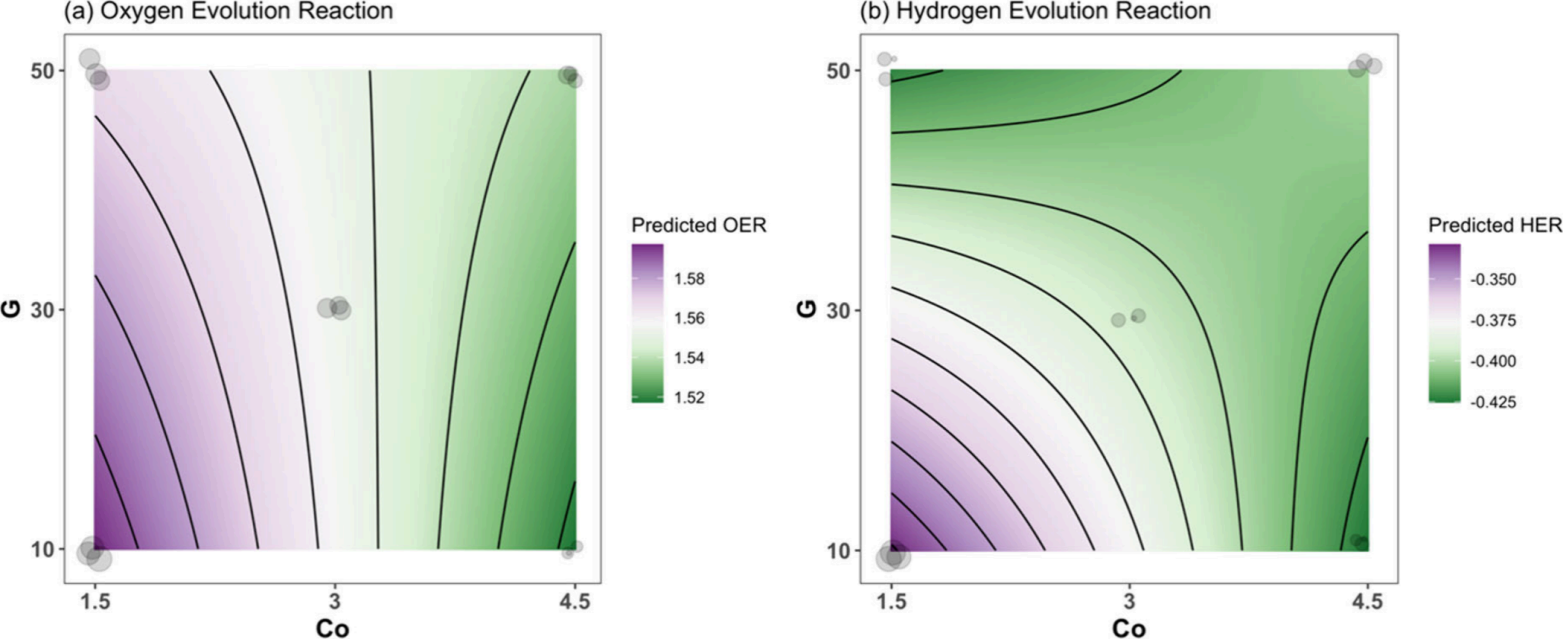
(a) Predicted OER and (b) HER across a 2D gradient of Co and G. Observed values are displayed as gray points, whose sizes are proportional to the observed value. Points are slightly jittered to ease visualization.

According to the equations presented in eq 1 and eq 2, a direct correlation is established between the considered factors and the response, revealing a response surface for potential across cobalt concentration and graphene amount gradients. The 2D contour plots depicted in Figure 2(a) and Figure 2(b) illustrate these primary and interaction effects, affirming the pronounced connection between the cobalt and graphene concentrations and onset potential. The optimal cobalt concentrations lie within the 4.0 to 4.5 mmol range, as delineated in the contour plot for the OER, and the optimum combinations with graphene amounts between 10 and 15 mg for the HER. Consequently, the Co_[4.5]_Cu_[3]_Fe_[3]_-LDH/G_[10]_ and Co_[1.5]_Cu_[3]_Fe_[3]_-LDH/G_[10]_ were chosen as the optimized material for the OER and HER, respectively, for further investigations. Additionally, control materials were prepared to elucidate the specific contributions of each component, facilitating a comprehensive understanding of their roles in influencing OER and HER activities.

### 3.2 Characterization of Optimized LDH Composites

Since the variations in cobalt quantities did not yield discernible distinctions in the characterization analyses, the Co_[4.5]_Cu_[3]_Fe_[3]_-LDH/G_[10]_ configuration was chosen for comprehensive characterization employing a blend of FE-SEM, XPS, XRD, and FTIR analyses.

**Figure 3:**
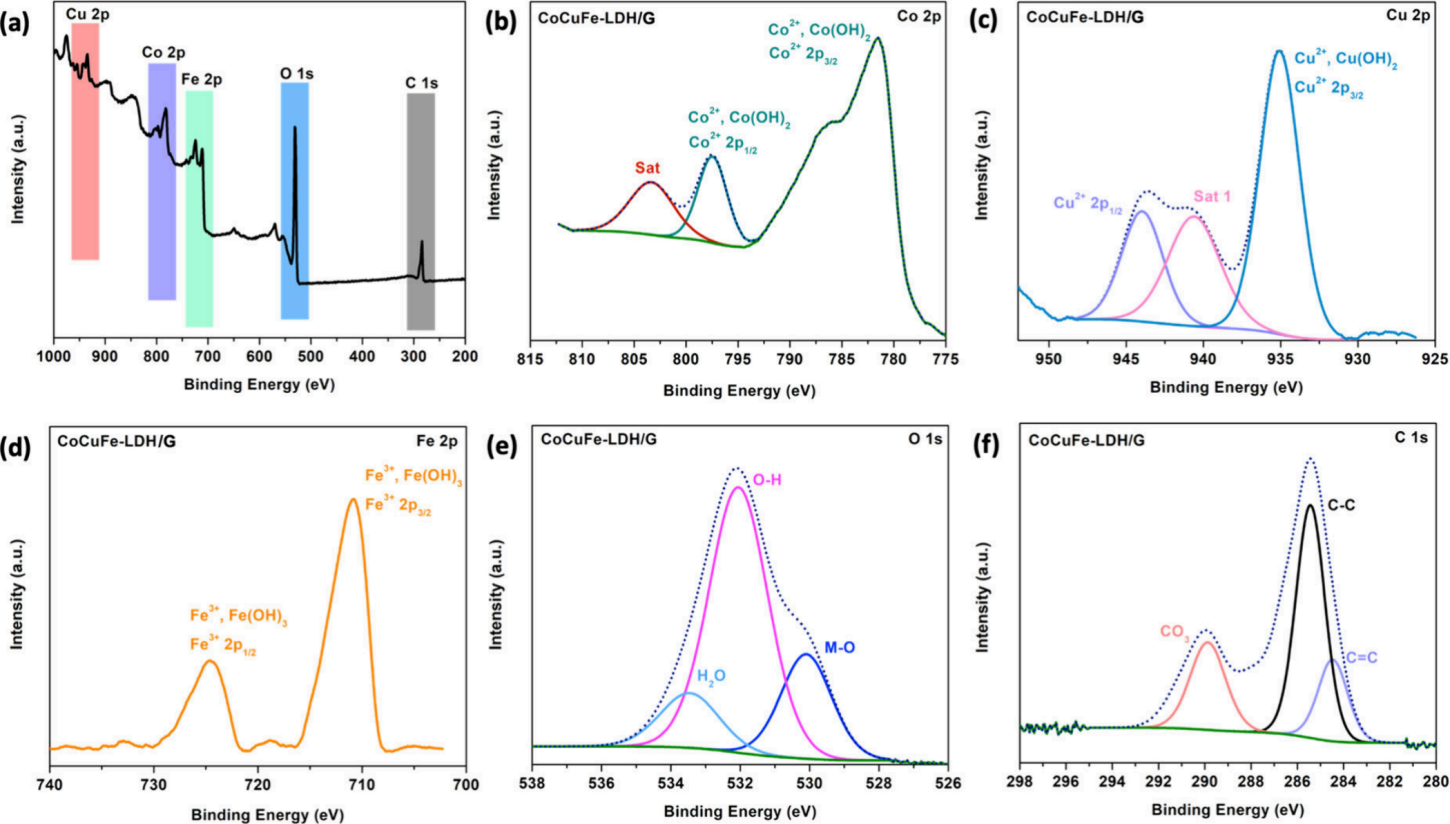
XPS of Co_[4.5]_Cu_[3]_Fe_[3]_-LDH/G_[10]_: (a) survey, (b) Co 2p, (c) Cu 2p, (d) Fe 2p, (e) O 1s, and (f) C 1s.

The chemical composition and valence states of the LDH composites were analyzed by XPS, as illustrated in Figure 3. The Co 2p spectra, in Figure 3(a), exhibit two distinct peaks at 781.6 and 797.5 eV, corresponding to Co^2+^ 2p_3/2_ and Co^2+^ 2p_1/2_ signals, respectively; these peaks are accompanied by one normal satellite peak at 803.2 eV.^38^ The spectra for Cu 2p presented in Figure 3(b) demonstrate characteristic peaks at 935.1 and 944.0 eV indicative of Cu^2+^ species within Cu(OH)_2_. These peaks are attributed to the Cu^2+^ 2p_3/2_ and Cu^2+^ 2p_1/2_ energy states, respectively. Furthermore, an accompanying satellite peak at 940.6 eV, corresponding to Cu^+^ 2p_1/2_, is observed, suggesting the formation of Cu_2_O.^39^ As evident in Figure 3(c), two peaks situated at 711.0 and 724.9 eV correspond to Fe^3+^ 2p_3/2_ and Fe^3+^ 2p_1/2_, respectively, which is consistent with previous works.^40,41^

In Figure 3(e), the O 1s spectrum shows two peaks positioned at 529.5 and 531.5 eV, corresponding to M–O and O–H, respectively, indicating the formation of hydroxyl interlayers ions in the trimetallic LDHs;^42^ whereas the peak at 533.2 eV is attributed to adsorbed water.^43^ The spectra for C 1s depicted in Figure 3(f) show two deconvoluted peaks at 284.5 and 285.4 eV which are associated with C=C and C–C bonds, respectively, indicating the successful introduction of graphene.^44^ Additionally, one peak at 289.5 eV is displayed in C the 1s spectra, corresponding to CO_3_ which can potentially originate from reaction byproducts,^45^ or indicate the presence of intercalated carbonate anions. The observed peaks in the spectra associated with these constituent elements indicate the achievement of successful synthesis of the intended heterogeneous architecture of the trimetallic LDH composites.

**Figure 4:**
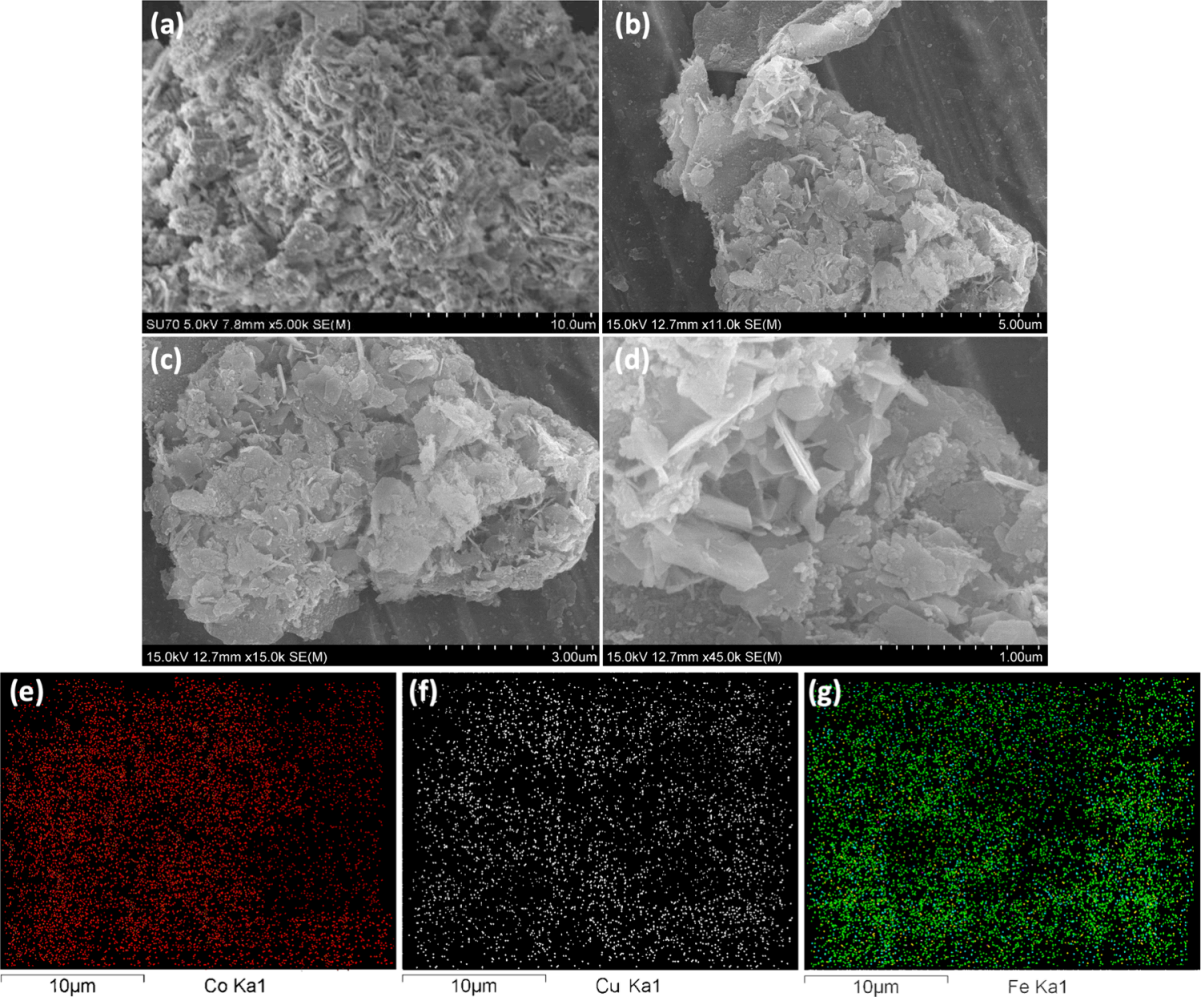
FE-SEM images of Co_[4.5]_Cu_[3]_Fe_[3]_-LDH/G_[10]_ at different magnifications with scales at (a) 10 μm, (b) 5 μm, (c) 3 μm, and (d) 1 μm; (e–g) elemental map of Co, Cu, and Fe, respectively.

The surface morphology is shown in Figure 4. The surface of CoCuFe-LDH/G exhibits nanoflakes decorated with small nanolayers and hierarchical architectures, culminating in the formation of clusters arranged vertically, as shown in Figure 4(a)-(b), consistent with recent experimental observations.^46^ In addition, the CoCuFe-LDH nanosheets are stacked layer by layer, being compactly arranged, which results in a dense surface coating (Figure 4(c)). The positively charged metal layer of CoCuFe-LDH interacts electrostatically with the negatively charged graphene nanoplates, facilitating their adsorption onto the CoCuFe-LDH surface. This interaction leads to the development of an LDH-G coating characterized by graphene nanoplatelets covering the surface—a distinctive feature of graphene.^38,47^ Furthermore, the sectional FE-SEM images of Co_[4.5]_Cu_[3]_Fe_[3]_-LDH/G_[10]_ in Figure 4(d) demonstrate close attachment and coverage of graphene on its surface. Additionally, Figure 4(e)-(g) illustrates the surface distribution of the three metal elements (Co, Fe, and Cu), revealing that these elements are uniformly distributed throughout the entire structure of the Co_[4.5]_Cu_[3]_Fe_[3]_-LDH/G_[10]_ composite.

**Figure 5:**
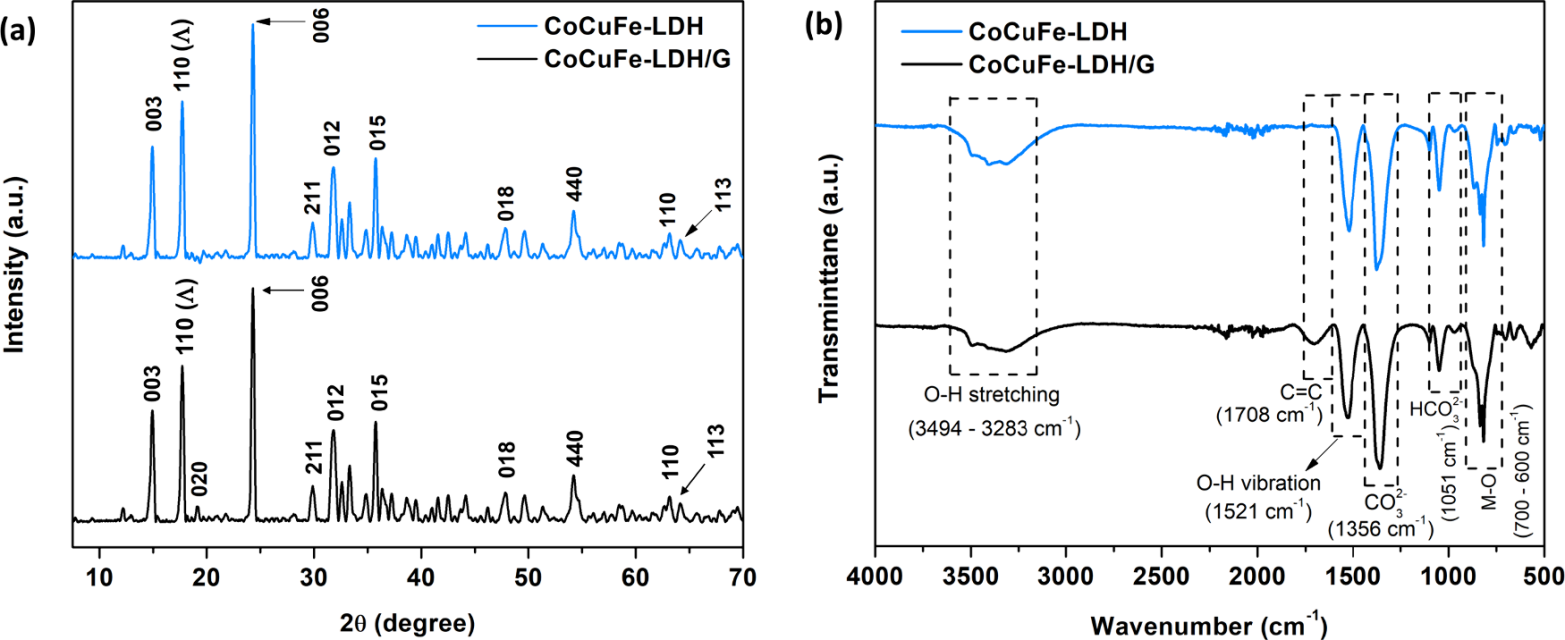
(a) XRD patterns, and (b) FTIR spectrum of Co_[4.5]_Cu_[3]_Fe_[3]_-LDH and Co_[4.5]_Cu_[3]_Fe_[3]_-LDH/G_[10]_.

XRD and FTIR analyses were conducted initially on the CoCuFe-LDH in the absence of graphene to gain information on the nature of the LDH. The crystalline nature of the LDHs was analyzed by XRD, and representative plots are illustrated in Figure 5(a). The XRD pattern of CoCuFe-LDH shows a series of diffraction peaks at 2θ = 14.7°, 24.6°, 33.4°, 37.9°, 47.1°, 59.9°, 62.6°, and 64.4°, corresponding to the (003), (006), (012), (015), (018), (440), (110), and (113) planes of Co(OH)_2_, Cu(OH)_2_ and Fe(OH)_2_, indicating the successful formation of the CoCuFe-LDH,^18,48^ in good agreement with the XPS analyses. Furthermore, the peaks at 17.8 and 29.9 correspond to (110 - Λ) and (211), respectively, which represent the HCO_3_^–^ orientation in the LDH.^45^ In the XRD analysis of the Co_[4.5]_Cu_[3]_Fe_[3]_-LDH/G_[10]_ composite, a similar diffraction pattern is observed, along with an additional weak peak at 19.8° which was attributed to the (020) plane of graphene,^46,47^ suggesting the formation of the Co_[4.5]_Cu_[3]_Fe_[3]_-LDH/G_[10]_ composite.

FTIR spectroscopy was employed to gain additional chemical information about the Co_[4.5]_Cu_[3]_Fe_[3]_-LDH/G_[10]_ composite and the corresponding CoCuFe-LDH in the absence of graphene. The FTIR spectra were recorded over the range of 4000 to 500 cm^–1^, as illustrated in Figure 5. A broad absorption band from 3494 to 3283 cm^–1^ is observed, indicative of the O–H stretching vibrations associated with interlayer H_2_O molecules, characteristic of LDHs.^49^ Furthermore, the vibrational modes of the CO_3_^2–^ and HCO_3_^–^ ions in the interlayer are identified at 1356 and 1051 cm^–1^, respectively.^50^ This is consistent with the XPS spectrum in Figure 3(f), which illustrates the presence of CO_3_^2–^. The bands observed in the range 700–600 cm^–1^ are attributed to the metal and oxygen lattice vibrations (M–O, M–O–M, and O–M–O) vibrations where M is Co, Cu and Fe.^16,51^ In comparison with CoCuFe-LDH, the FT-IR spectrum of the Co_[4.5]_Cu_[3]_Fe_[3]_-LDH/G_[10]_ composite reveals an additional weak absorption band at 1708 cm^–1^ associated with C=C vibrations in graphene,^52,53^ confirming successful incorporation of graphene nanoplatelets into the LDH structure.

### 3.3 Electrocatalytic Performance for Water Splitting

**Figure 6:**
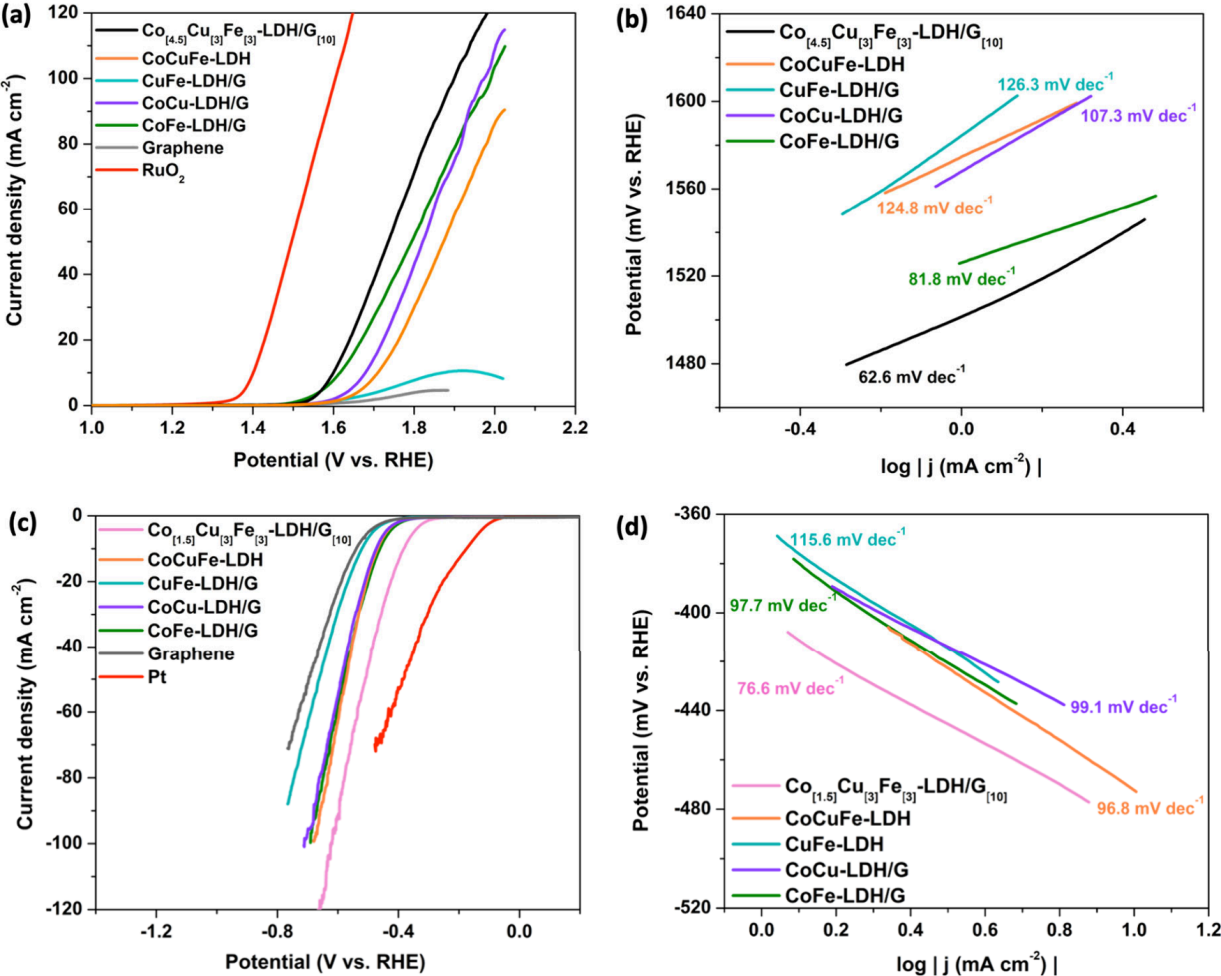
(a) OER polarization curves of the control materials and (b) Tafel slopes; (c) HER polarization curves of the control materials and (d) Tafel slopes.

Following the successful synthesis, optimization, and characterization of the Co_[4.5]_Cu_[3]_Fe_[3]_-LDH/G_[10]_, and Co_[1.5]_Cu_[3]_Fe_[3]_-LDH/G_[10]_, experiments were conducted to evaluate the electrocatalytic properties of the control materials. This investigation aimed to verify the impact of each component on the electrochemical performance, focusing on both OER and HER activity, respectively. Figure 6 shows the LSV curves and Tafel slopes for the OER and HER. For comparison, the electrocatalytic performance of RuO_2_ and Pt benchmark catalysts was also measured. The optimized Co_[4.5]_Cu_[3]_Fe_[3]_-LDH/G_[10]_ exhibits the lowest onset potential at 1.52 V (Figure 6(a)) and Tafel Slope of 62.6 mV dec^–1^ (Figure 6(b)) for OER compared to the control materials. Although the benchmark catalyst RuO_2_ exhibits a lower onset potential of 1.38 V, the optimized Co_[4.5]_Cu_[3]_Fe_[3]_-LDH/G_[10]_ remains promising with a near onset potential and similar curve slope. Meanwhile, the Pt provides an onset potential of −0.1 V for HER, as shown in Figure 6(c), indicating that the noble metal has a better HER performance compared to the LDH composites; however, the Co_[1.5]_Cu_[3]_Fe_[3]_-LDH/G_[10]_ shows promising catalytic performance with an onset potential around −0.32 V and Tafel slope of 76.6 mV dec^–1^ (Figure 6(d)). The CuFe-LDH/G displays the highest onset potential at 1.67 V with a Tafel slope of 126.3 mV dec^–1^ and −0.45 V with a Tafel slope of 115.6 mV dec^–1^ for the OER and HER, respectively, indicating that Co plays an important role in the performance of the OER and HER. As mentioned earlier, this can be attributed to the Co charge transfer effect, facilitating the oxidation of Co^2+^ to Co^3+^ in the OER.^17^ The Cu component in CoCuFe-LDH/G exhibits specific proton and hydrogen adsorption properties relevant to OER and HER, respectively;^18^ the incorporation of Fe and Co adjusts the binding energy of the Cu sites, thereby enhancing the OER and HER activities of CoCuFe-LDH/G compared to other bimetallic LDHs.^48^ Also, the presence of Fe^3+^ further promotes the oxidation of Co^2+^ to Co^3+^, resulting in CoOOH formation, which augments the OER activity.^19^ Clearly the combination of Co, Cu and Fe enhances the OER activity and this is consistent with recent reports describing high entropy electrocatalysts, which are very effective in water splitting.^54^ Theoretical and experimental findings have also shown that metals with different valence electron configurations play a crucial role in modulating electronic structures, driven by electron donor/acceptor interactions between the metal ions.^55^ Adjusting the electronic structure can alter the valence states of metal ions, significantly impacting catalytic performance.^56^ This is also relevant in the case of the LDHs. The incorporation of Fe^3+^ increases the electrical conductivity of the material and enhances the adsorption capability for reaction intermediates during the OER and HER.^23^ The Cu^2+^ acts as a modulator of the electronic structure, creating an electronic transfer channel and forming a strong electron coupling with CoFe-LDH/G in the CoCuFe-LDH/G system.^48^ This optimizes the electron cloud density at active sites, improves the adsorption of reaction intermediates, and enhances the intrinsic activity of OER and HER.^57^

Moreover, Co_[4.5]_Cu_[3]_Fe_[3]_-LDH/G_[10]_ and Co_[1.5]_Cu_[3]_Fe_[3]_-LDH without graphene exhibit a higher onset potential at 1.62 V and a Tafel slope of 124.8 mV dec^–1^ for the OER and −0.50 V with a Tafel slope of 96.8 mV dec^–1^ for the HER compared to the composites with graphene. This suggests that the conductive nanoflake structure established by graphene facilitates a sufficient electron supply to the composite during the electrocatalytic process.^27^ The observed effect can also be attributed to the large surface area of the nanoflakes adorned with fine nanolayer structures, according to the SEM images in Figure 4, combined with the absence of a binder between the LDH and graphene.^58^ This configuration enhances the surface reaction and facilitates direct contact between the nanolayers and the underlying conductive substrate, ensuring that each nanolayer actively participates in the reaction;^59^ thereby enhancing OER and HER activities.

Further insights into the kinetics and mechanism of the electrochemical reactions were obtained using Tafel analysis. The corresponding Tafel slopes derived from the LSV curves are shown in Figure 6(b) and Figure 6(d), for the OER and HER, respectively. The Tafel slope (*b*) determines how fast the current density (*i*) increases as a function of overpotential (η) and can be calculated according to the equation below:
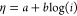
3The Co_[4.5]_Cu_[3]_Fe_[3]_-LDH/G_[10]_ exhibited a low Tafel slope of 62.8 mV dec^–1^, while Co_[1.5]_Cu_[3]_Fe_[3]_-LDH/G_[10]_ showed a Tafel slope of 76.6 mV dec^–1^. This, in conjunction with the observed low onset potential compared to other LDHs, suggests that the optimized composite possesses an electronic structure conducive to more efficient adsorption/desorption of oxygenated species, thereby enhancing electrocatalytic activity for both the OER and HER. Furthermore, the CoCuFe-LDH without graphene shows higher Tafel slopes, approximately 124.8 and 96.8 mV dec^–1^ for the OER and HER, respectively, underscoring the notable role of graphene in enhancing the electrocatalytic performance of the composites.

Based on a comparison with other composites in the literature, as illustrated in Table 4, the Co_[m]_Cu_[3]_Fe_[3]_-LDH/G_[n]_ composites developed in this work show a lower overpotential at 10 mA cm^–2^. This indicates that the electrocatalytic performance of this composite is better or comparable to that of other reported electrocatalysts for the OER. This can be attributed to the *in situ* growth of CoCuFe-LDH on the graphene substrate, which effectively prevents the restacking of the graphene sheets while helping to prevent aggregation of the LDHs, leading to strong interactions and synergistic effects between the CoCuFe-LDH and the graphene. These effects accelerate electron transfer, resulting in improved electrocatalytic performance for both OER and HER.

**Table 4:** Performance Comparison of the Optimized LDH with the Literature

**Composites**	**Electrolyte**	**Overpotential at 10mA cm**^**–2**^**(mV)**	**Ref**
**OER**			
CoAl-LDH/NG	1.0 M KOH	365	(60)
NiFe-LDH/Ni–Fe phosphide	1.0 M KOH	350	(61)
CoAl-LDH	1.0 M KOH	415	(60)
CO_3_–CoAl-LDH	1.0 M KOH	422	(62)
CuCo_2_O_4_@CuCoNi-LDH	1.0 M KOH	330	(63)
ZnCo-LDH	1.0 M KOH	420	(64)
Co_[4.5]_Cu_[3]_Fe_[3]_-LDH/G_[10]_	1.0 M KOH	350	This work
**HER**			
Pt–Co/Fe-LDH	1.0 M KOH	360	(65)
NiFe-LDH	1.0 M KOH	530	(61)
CoNi@NiFe-LDH	1.0 M KOH	380	(66)
NiCo_2_O_4_ on Ni foam	1.0 M KOH	394	(67)
NiFe LDH-NS/G_[10]_^a^	1.0 M KOH	490	(68)
Co_[1.5]_Cu_[3]_Fe_[3]_-LDH/G_[10]_	1.0 M KOH	380	This work

a NS: nanosheets.

### 3.4 Electronic Conductivity and Stability Characteristics

**Figure 7:**
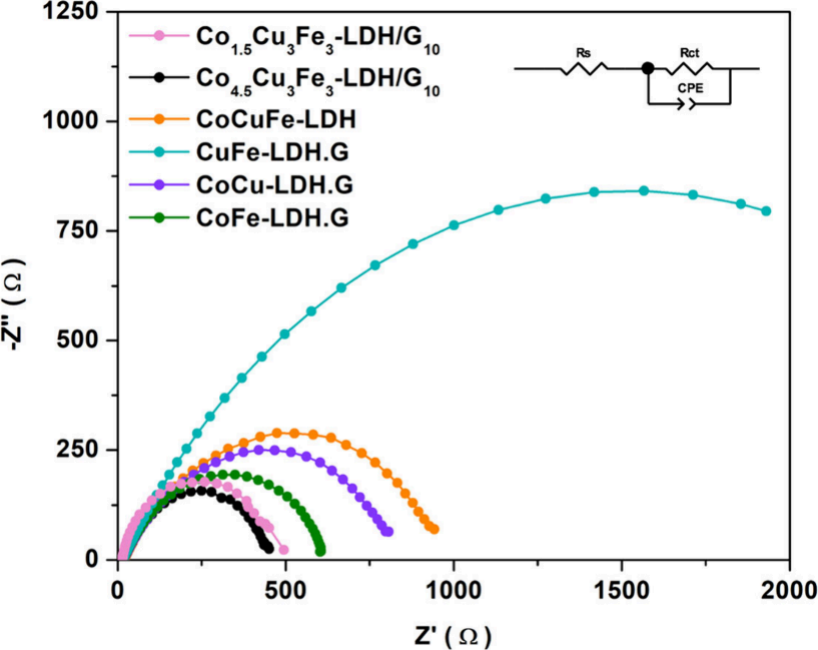
Electrochemical impedance for the control materials (the equivalent circuit model is shown as inset). Data were collected across a frequency range from 1 × 10^6^ to 0.007 Hz using fixed potentials at 1.59 V and −0.41 V, which correspond to 10 mA cm^–2^ for OER and HER, respectively. HER data show a pink trace using the Co_[1.5]_Cu_[3]_Fe_[3]_-LDH/G_[10]_.

Additional information about the electronic conductivity of the composites was obtained through electrochemical impedance spectroscopy (EIS). Figure 7 illustrates the equivalent circuits utilized for fitting the impedance data, comprising solution resistance (*R*_s_), charge transfer resistance (*R*_CT_) and a constant phase element (CPE). The impedance profiles of the optimized Co_[4.5]_Cu_[3]_Fe_[3]_-LDH/G_[10]_ and Co_[1.5]_Cu_[3]_Fe_[3]_-LDH/G_[10]_ composites align with a simplified Randles cell model. The plots are characterized by a depressed semicircle, as shown in Figure 7. The diameter of the semicircle, reflecting the *R*_CT_, exhibits notable variation depending on the LDHs nature, distinctly indicating that the Co_[4.5]_Cu_[3]_Fe_[3]_-LDH/G_[10]_ and Co_[1.5]_Cu_[3]_Fe_[3]_-LDH/G_[10]_ composites have the lowest charge transfer resistance. Furthermore, given the comparable electronic properties of the optimized composites, Figure 7 presents the EIS for the control materials employing 4.5 mmol of cobalt.

The comparison of the computed R_CT_ values is presented in Table 5. Notably, both Co_[4.5]_Cu_[3]_Fe_[3]_-LDH/G_[10]_ and Co_[1.5]_Cu_[3]_Fe_[3]_-LDH/G_[10]_ exhibit the smallest charge transfer resistance, measured at 486.60 ± 15.69 and 500.00 ± 14.58 Ω, respectively, aligning with their lower onset potential and Tafel slopes. On comparing the bimetallic LDHs, CoFe-LDH/G, CoCu-LDH/G, and CoFe-LDH/G, it is evident that the presence of Co leads to a considerable reduction in the charge transfer resistance, with CoFe-LDH/G having the lowest *R*_CT_ of 637.80 ± 14.65 Ω and CuFe having the highest at 3024.00 ± 174.40 Ω. However, this is mainly connected with the rate of the OER, with the Co-containing LDHs possessing the highest OER activity and therefore the lowest R_CT_, while the Co-free LDHs have a much lower OER activity, as seen in Figure 6(a). Moreover, the CoCuFe-LDH without graphene shows a higher charge transfer resistance of 1057.00 ± 38.92 Ω. Therefore, the introduction of graphene in Co_[4.5]_Cu_[3]_Fe_[3]_-LDH/G_[10]_ and Co_[1.5]_Cu_[3]_Fe_[3]_-LDH/G_[10]_ enhances the electronic conductivity, thereby accelerating the charge transfer rate in both the OER and HER processes.

**Table 5:** Electrochemical Impedance Parameters

**Composites**	***R***_**CT**_**(Ω)**	**CPE (F)**
Co_[1.5]_Cu_[3]_Fe_[3]_-LDH/G_[10]_	500.00 ± 14.58	7.02 × 10^–4^
Co_[4.5]_Cu_[3]_Fe_[3]_-LDH/G_[10]_	486.60 ± 15.69	6.34 × 10^–4^
CoCuFe-LDH	1057.00 ± 38.92	12.54 × 10^–4^
CuFe-LDH/G	3024.00 ± 174.40	16.41 × 10^–4^
CoCu-LDH/G	906.50 ± 31.76	14.05 × 10^–4^
CoFe-LDH/G	637.80 ± 14.65	8.59 × 10^–4^

The capacitance of the LDHs is relatively high in the vicinity of 1 mF cm^–2^, which is consistent with the presence of the Fe, Co, and Cu cations, the OH^–^ ions and the high surface area seen in Figure 4. Interestingly, it was observed that the optimized composites exhibit the lowest capacitance, as shown in Table 4, compared to the control materials. This may indicate a somewhat lower surface area for the optimized composites; however, with a lower *R*_ct_, these optimized composites are clearly more promising electrocatalysts.

**Figure 8:**
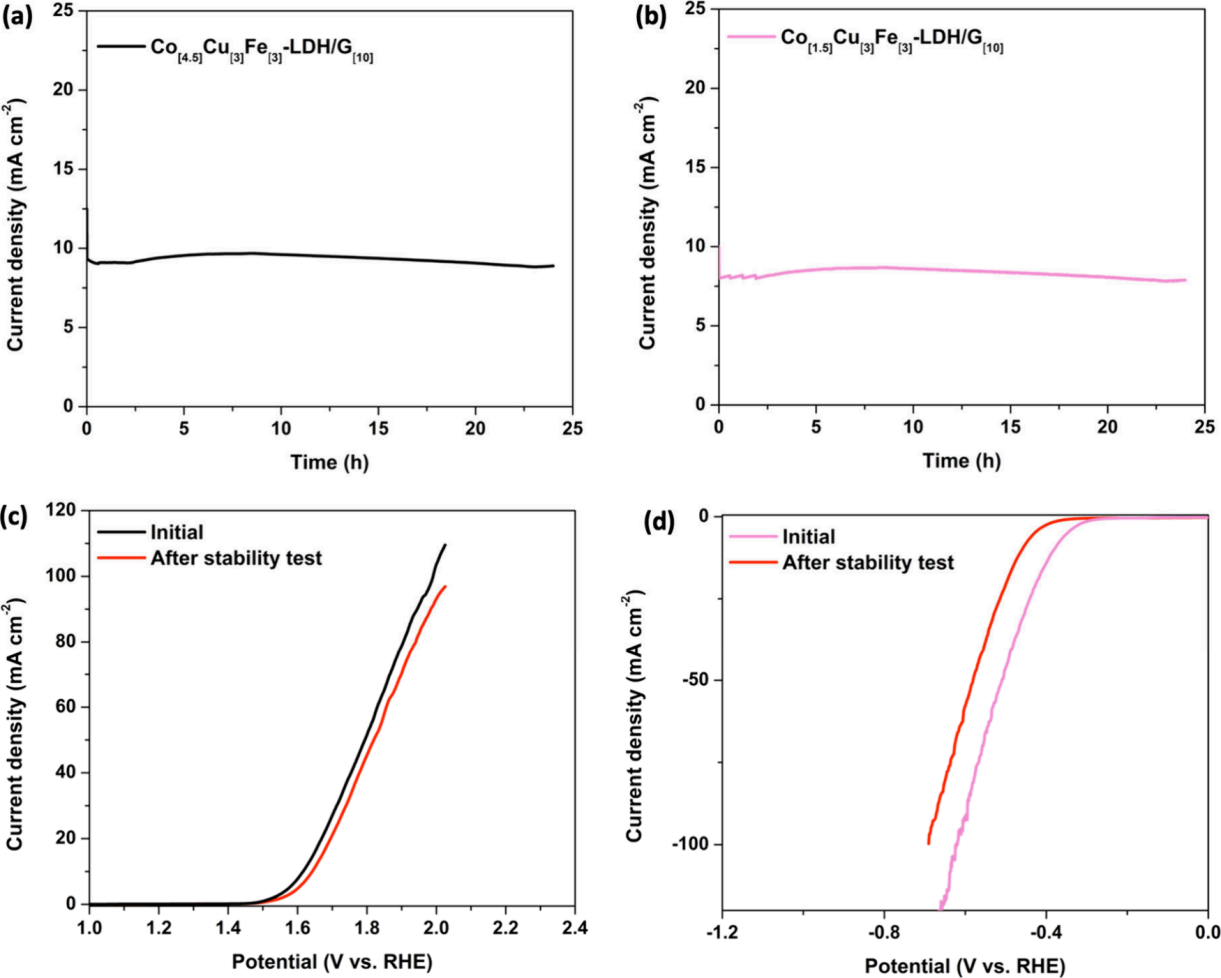
Long-term stability test of (a) Co_[4.5]_Cu_[3]_Fe_[3]_-LDH/G_[10]_, and (b) Co_[1.5]_Cu_[3]_Fe_[3]_-LDH/G_[10]_ at 10 mA cm^–2^; polarization curves before and after stability test for (c) the OER and (d) the HER, respectively.

The pivotal role of stability in determining the practical applicability of electrocatalysts is widely acknowledged. Consequently, the stability of the optimized Co_[4.5]_Cu_[3]_Fe_[3]_-LDH/G_[10]_ and Co_[1.5]_Cu_[3]_Fe_[3]_-LDH/G_[10]_ composites was investigated through a chronoamperometry experiment, employing a fixed potential (1.59 V and −0.41 V for OER and HER, respectively) corresponding to a current density of 10 mA cm^–2^. The resultant current–time plots are presented in Figure 8(a) and Figure 8(b), revealing a slight decrease in current density to 9.56 mA cm^–2^ and 8.67 mA cm^–2^, for Co_[4.5]_Cu_[3]_Fe_[3]_-LDH/G_[10]_ and Co_[1.5]_Cu_[3]_Fe_[3]_-LDH/G_[10]_, respectively, after 24 h due to the adsorption of the generated bubbles on the active sites.

The negligible degradation in current density over an extended period affirms the advantageous durability and catalytic persistence of both Co_[4.5]_Cu_[3]_Fe_[3]_-LDH/G_[10]_ and Co_[1.5]_Cu_[3]_Fe_[3]_-LDH/G_[10]_. Further support for their stability is evident in the minimal differences observed in the LSV curves in Figure 8(c) and Figure 8(d) for the OER and HER, respectively, before and after the stability test, particularly at lower current densities. Therefore, both composites demonstrate good stability without significant loss of catalytic activity over 24 h.

**Figure 9:**
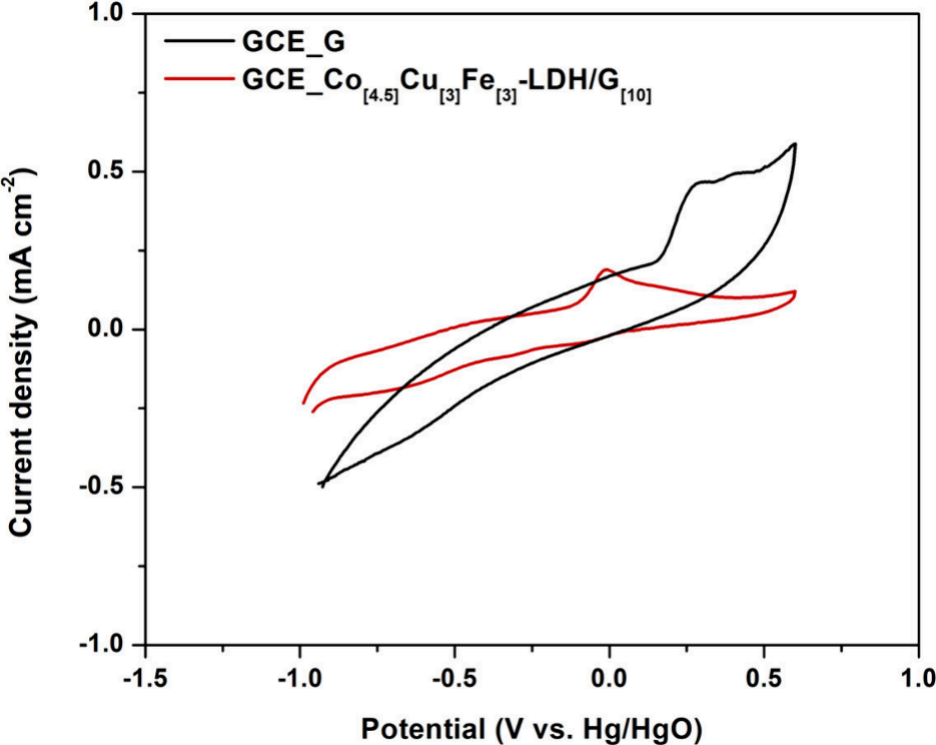
Cyclic voltammetry in buffer (pH 7.0) of a glassy carbon electrode (GCE) with graphene nanoplatelets and GCE with Co_[4.5]_Cu_[3]_Fe_[3]_-LDH/G_[10]_.

Clearly both the OER and the HER are facilitated by the graphene sheets dispersed throughout the composite. However, graphene sheets can oxidize in an alkaline environment when subjected to high potentials necessary for the OER. To evaluate the stability of the graphene nanoplatelets both independently and within the layered double hydroxide (LDH) composite, the material was immersed in a 1 M KOH solution and subjected to electrochemical testing. This stability test involved maintaining a fixed potential of 0.50 V (V vs Hg/HgO) for 1 h, followed by an assessment of graphene oxidation using cyclic voltammetry (CV) in a phosphate buffer solution at pH 7.0, with a potential range typical for graphene oxidation. The CV curves demonstrated consistent behavior over 50 cycles, therefore, the final cycle for both tests was plotted, as shown in Figure 9. The two plots are very different. The peak at about 0.0 V corresponds to the oxidation of Co in the LDH.^69^ A peak at 0.4 V signifies the oxidation of graphene, while a weak peak at about −1.0 V can be associated with the reduction of the oxygenated species generated at high potentials.^70^ The absence of this peak at 0.4 V in the Co_[4.5]_Cu_[3]_Fe_[3]_-LDH/G_[10]_ CV profile suggests that graphene maintained its structural integrity throughout the testing period when incorporated into the composite. This stability can be attributed to the presence of the LDH, which appears to protect the graphene from oxidation at least during short-term testing.

## 4 Conclusions

In this study, ternary composites of Co_[m]_Cu_[3]_Fe_[3]_-LDH/G_[n]_ were synthesized via a one-step hydrothermal process. The effect of the concentration of cobalt and graphene on the OER and HER performance was investigated by a 2-level full factorial design to optimize the LDH composite for water electrolysis. The Co_[4.5]_Cu_[3]_Fe_[3]_-LDH/G_[10]_ and Co_[1.5]_Cu_[3]_Fe_[3]_-LDH/G_[10]_ composites reached the lowest onset potential of 1.52 V and −0.32 V, for OER and HER, respectively. This indicates that a high concentration of cobalt enhances OER activity, while a lower concentration improves the HER performance; on the other hand, an optimal concentration of graphene for both OER and HER was observed at a lower concentration of 10 mg. Additionally, statistical analysis revealed a significant interaction between Co and G for both the OER and HER. Furthermore, the optimized composites demonstrated favorable electronic properties and stability, maintaining significant catalytic activity over 24 h. Therefore, this study provides a new insight for a facile and efficient strategy to design and optimize a trimetallic LDH electrocatalyst combined with graphene, demonstrating that the conductive nanoflake structure established by graphene provides a sufficient electron supply to the composite during the electrocatalytic process, enhancing the OER and HER activities.
